# Motor imagery ability in baseball players with throwing yips

**DOI:** 10.1371/journal.pone.0292632

**Published:** 2023-11-30

**Authors:** Toshiyuki Aoyama, Kazumichi Ae, Hiroto Soma, Kazuhiro Miyata, Kazuhiro Kajita, Takashi Kawamura

**Affiliations:** 1 Department of Physical Therapy, Ibaraki Prefectural University of Health Sciences, Ami-Machi, Inashiki-gun, Ibaraki-ken, Japan; 2 Faculty of Sport Science, Nippon Sport Science University, Tokyo, Japan; 3 Department of Sports Rehabilitation, Gakusai Hospital, Nakagyo-ku, Kyoto-city, Kyoto-fu, Japan; 4 Department of Health and Sport Sciences, Faculty of Health and Medical Sciences, Kyoto University of Advanced Science (KUAS), Sogabe, Kameoka-city, Kyoto-fu, Japan; 5 Institute of Health and Sport Sciences, University of Tsukuba, Tsukuba-city, Ibaraki-ken, Japan; Universita degli Studi di Torino, ITALY

## Abstract

The motor imagery ability is closely related to an individual’s motor performance in sports. However, whether motor imagery ability is diminished in athletes with yips, in whom motor performance is impaired, is unclear. Therefore, this cross-sectional study aimed to determine whether general motor imagery ability or vividness of motor imagery specific to throwing motion is impaired in baseball players with throwing yips. The study enrolled 114 college baseball players. They were classified into three groups: 33 players in the yips group, 26 in the recovered group (previously had yips symptoms but had resolved them), and 55 in the control group. They answered the revised version of the vividness of movement imagery questionnaire (VMIQ-2), which assesses general motor imagery ability. Furthermore, they completed a questionnaire that assesses both positive and negative motor imagery vividness specific to baseball throwing. In the former, they responded to their ability to vividly imagine accurately throwing a controlled ball, whereas in the latter, they responded to the vividness of their experience of negative motor imagery associated with baseball throwing, specifically the image of a wild throw. No significant difference in the VMIQ-2 was found among the three groups. While no significant difference in the vividness of positive motor imagery for ball throwing was found in either first-person visual or kinesthetic perspectives among the three groups, the yips group exhibited significantly higher vividness of negative motor imagery than the control group in both perspectives. These results indicate that negative motor imagery specific to baseball throwing may be associated with symptoms of yips. Therefore, interventions addressing psychological aspects, such as anxiety, which are potential causes of the generation of negative motor imagery, may be necessary to alleviate the symptoms of yips.

## Introduction

The yips is a psychoneuromuscular disorder characterized by involuntary movements that interfere with the execution of coordinated motor action in sports [1, 2]. In golfers’ yips, they experience symptoms such as freezing, jerks, jitters, and twitches during their putting motion, resulting in impaired putting skills [3, 4]. Although yips have been reported to occur not only in golf but also in various other sports such as archery [5], darts [6], cricket [2], and baseball [7]. In a study on baseball players, we reported that the occurrence of yips was associated with players’ personality traits [7]. Furthermore, a high percentage (47%) of college baseball players reported having experienced yips symptoms, causing a serious impact on their careers [8]. Therefore, effective treatment methods must be developed to alleviate its symptoms.

A potential intervention for yips is motor imagery, which involves the mental simulation of a given movement without physical output [9, 10]. Numerous neurophysiological and neuroimaging studies [11–15] have shown that neural activity during motor imagery is similar to that during actual motor execution. Based on this neurophysiological evidence, interventions using motor imagery have been effective in improving muscle strength [16] and motor skills [17–19]. Similarly, studies on athletes reported that interventions using motor imagery improve sports performance [20, 21]. Furthermore, such interventions are effective in the rehabilitation of injured athletes [22, 23]. In addition, in focal dystonia, a yips subtype, interventions using motor imagery are reported to reduce its symptoms [24, 25]. Bell et al. have also examined the effects of interventions using motor imagery techniques on yips-affected athletes [26, 27]. Although their studies were case studies with small sample size, they reported a decrease in yips symptoms. Thus, future large-scale studies on the effects of motor imagery are warranted.

Motor imagery ability can be assessed using questionnaires. The revised version of the Motor Imagery Questionnaire (MIQ) [28, 29] is one of the most commonly used measures of motor imagery ability. The MIQ-revised second edition is a valid and reliable measure of imagery ability for basic upper and lower limbs and trunk movements [28]. The revised version of the vividness of movement imagery questionnaire (VMIQ-2) [30] measures how vividly people can imagine themselves performing 12 movements with high construct and concurrent validity. Given that VMIQ-2 includes items required for sports movements, such as jumping, running, and kicking, it is suitable for assessing motor imagery ability in athletes [30, 31]. The motor imagery ability is closely related to an individual’s motor skills [32, 33]. For example, Corrado et al. revealed that competitive athletes had higher scores on motor imagery ability than non-athletes [33]. Conversely, the motor imagery ability can be impaired by neurological [34, 35] or musculoskeletal [36, 37] disorders. Therefore, it is conceivable that motor imagery ability is similarly diminished in yips-affected athletes with impaired motor skills; however, no studies have evaluated this possibility. Furthermore, whether impaired imagery ability in athletes with yips occurs in general limb movements or sport-specific movements is unclear. Given these backgrounds, this study aimed to determine whether general motor imagery ability and vividness of motor imagery specific to throwing motion are impaired in baseball players with throwing yips. Given that yips symptoms are movement-specific [38], we hypothesized that athletes with throwing yips may have impairments in motor imagery ability specific to ball throwing.

## Materials and methods

### Participants

This cross-sectional study included the following three participant groups: those who still have yips symptoms (yips group), those who had yips symptoms in the past that are now resolved (recovered group), and those who never had yips symptoms (control group). The sample size was calculated using G*Power, with a power of 0.8, an α of 0.05, and effect size η2 = 0.14. As a result, 22 players were required per group. In addition, based on a previous study [3] and our preliminary research (unpublished data), we estimated that a total of 110 participants would be required since the expected proportion of players with yips or recovering from them was one-third of those without yips. E-mail invitation to participate in this study was sent to the players of a team belonging to the First Division of the Tokyo Metropolitan Area Collegiate Baseball League from 2019 to 2022. Among them, 117 players participated in this study of their own accord. The participants had an average (standard deviation) age of 20.3 (1.3) years and an average of 12.5 (2.1) years of baseball experience. In this study, the yips was defined as a state in which symptoms such as repeated wild throws cannot be caught by the throwing partner for more than 1 month, similar to the criteria used in our previous study [7]. Three players who had experienced yips symptoms but for less than 1 month were excluded from the study [7]. As a result, 33 players were included in the yips group, 26 in the recovered group, and 55 in the control group (Table 1). None of the participants had any specific experience in motor imagery training.

**Table 1:** Participant characteristics.

	Control	Yips	Recovered	p-value
Number of players (n)	55	33	26	―
Age (years)	20.0 (19.0, 21.0)	21.0 (20.0, 21.0)	20.5 (20.0, 21.0)	0.252
Throwing side (R/L)	47/8	30/3	25/1	0.347
Baseball experience (years)	12 (11, 14)	13 (11, 14)	12 (11, 14)	0.608
Position (P/C/IF/OF)	18/11/15/11	7/9/8/9	7/9/10/0	0.117

The Kruskal–Wallis test was used to analyze age and baseball experience: median (first, third quartile)

The Fisher–Freeman–Halton test was used for the throwing arm: n

The Chi–square test was used for the position: n

P, pitcher; C, catcher; IF, infielder, OF, outfielder

This study was performed in accordance with the recommendations of the Declaration of Helsinki established by the World Medical Association. The protocol was approved by the local ethics committee of the Ibaraki Prefectural University of Health Sciences (Approved no. 876), and written informed consent for participation in this study was obtained from all participants. During or after data collection, the authors did not access any information that could identify individual participants.

### Questionnaires

The participants answered an anonymous self-administered questionnaire. Once completed, they submitted the questionnaire to a researcher who was not affiliated with the players. Each player answered a questionnaire regarding their motor imagery ability. The VMIQ-2 [30] was used to assess the general motor imagery ability. Previous studies have shown that the VMIQ-2 has factorial, concurrent, and construct validity [30]. Data for two factors of the VMIQ-2 were collected: first-person visual imagery and kinesthetic imagery. Each of them contains a 12-item assessment of motor imagery ability, such as running, jumping, throwing, and kicking. Each item consists of a 5-point Likert scale, ranging from 1 to 5, with lower values reflecting higher motor imagery ability. Thus, in each category, a score of 12 points means the highest ability in motor imagery; conversely, a score of 60 points means the worst.

As for the content of motor imagery vividness, participants can imagine movements, not only in positive directions, such as good motor performance, but also in negative ones, such as poor motor performance, i.e., failure of sports-related movements [39–41]. Therefore, a visual analog scale (VAS) (0–100 mm) [42, 43] was used to investigate the vividness of positive and negative motor imagery specific to baseball throwing. In the question regarding positive motor imagery vividness, each player was asked “How clearly can you imagine the visual image (or kinesthetic sensation) in your head that you are throwing a precisely controlled ball toward a target?” The left (0 mm) and right (100 mm) ends of the VAS indicated “I could not imagine the visual image (or kinesthetic sensation) at all” and “I could imagine the visual image (or kinesthetic sensation) as if it were the actual image (or kinesthetic sensation),” respectively. In the question regarding negative motor imagery vividness, the players were asked “When you imagine the first-person visual image (or kinesthetic sensation) of yourself throwing a ball in your head, do you tend to imagine a negative visual image (or kinesthetic sensation) that you will make a wild throw?” The left (0 mm) and right (100 mm) ends of the VAS indicated “strongly disagree” and “strongly agree,” respectively.

### Analysis

All statistical analyses were performed using SPSS26 (IBM Corp., USA) with a significance level of p = 0.05. Since the VMIQ-2 includes an item related to ball throwing, for the item “Throwing a stone into water,” we had to perform an independent statistical analysis in addition to the VMIQ-2 total score. Normality and homoscedasticity assumptions were tested using the Shapiro–Wilk and Levene’s tests, respectively, for all variables. Since the variables used in all statistical comparisons included those for which normality and homoscedasticity assumptions were not met, a nonparametric Kruskal–Wallis test (comparison among the control, yips, and recovered groups) was performed. When significant p values were obtained, a Mann Whitney U-test with Bonferroni correction was conducted for multiple comparisons. The effect size η^2^ was also calculated. The effect size was defined as small for η^2^ > 0.01, medium for η^2^ > 0.06, and large for η^2^ > 0.14 [44]. Spearman’s rank correlation coefficient was calculated to investigate the association between positive and negative motor imagery vividness specific to ball throwing. Data are presented as median (first and third quartiles).

## Results

The raw dataset is shown in S1 Table. The results of the VMIQ-2 and the throwing-specific motor imagery vividness are shown in Table 2. The VMIQ-2 first-person visual scores were 20.0 (14.0, 28.0) in the control group, 21.0 (12.5, 30.5) in the yips group, and 21.5 (13.0, 26.5) in the recovered group. In the Kruskal–Wallis test, no significant difference was found in the VMIQ-2 first-person visual scores (Z [2] = 0.004, p = 0.998, η^2^ = 0.018). The VMIQ-2 kinesthetic scores were 23.0 (18.0, 28.0) in the control group, 23.0 (13.0, 29.0) in the yips group, and 22.0 (18.8, 29.0) in the recovered group, and the difference was not statistically significant (Z [2] = 0.335, p = 0.846, η^2^ = 0.015). There were no significant differences for “Throwing a stone into water,” an item on the VMIQ-2, from both first-person visual (Z [2] = 0.009, p = 0.995) and kinesthetic (Z [2] = 0.697, p = 0.706) perspectives.

**Table 2:** Results of the questionnaire.

	Perspective	Control	Yips	Recovered	p-value	η^2^	Post hoc test (p < 0.05)
VMIQ-2	First-person visual	20.0 (14.0, 28.0)	21.0 (12.5, 30.5)	21.5 (13.0, 26.5)	0.998	0.018	―
	Kinesthetic	23.0 (18.0, 28.0)	23.0 (13.0 29.0)	22.0 (18.8, 29.0)	0.846	0.015	―
Positive motor imagery vividness	First-person visual	84.4 (67.2, 100)	74.6 (51.2, 87.4)	82.4 (65.0, 88.7)	0.082	0.027	―
	Kinesthetic	85.2 (67.2, 99.2)	73.8 (49.2, 91.8)	82.0 (67.6, 93.0)	0.077	0.028	―
Negative motor imagery vividness	First-person visual	24.6 (12.3, 53.3)	73.8 (37.0, 91.8)	45.1 (17.8, 68.6)	<0.001	0.190	Control vs. yips
	Kinesthetic	24.6 (7.4, 58.2)	65.6 (44.7, 88.6)	49.2 (24.9, 75.2)	<0.001	0.160	Control vs. yips

The Kruskal–Wallis test was used for all statistical analysis: median (first and third quartiles)

VMIQ-2, revised version of the vividness of movement imagery questionnaire

The vividness scores of throwing-specific positive motor imagery in the first-person visual perspective were 84.4 (67.2, 100) in the control group, 74.6 (51.2, 87.4) in the yips group, and 82.4 (65.0, 88.7) in the recovered group. In the Kruskal–Wallis test, no significant differences were found among the three groups (Z [2] = 4.998, p = 0.082, η^2^ = 0.027). The vividness scores of throwing-specific positive motor imagery in the kinesthetic perspective were 85.2 (67.2, 99.2) in the control group, 73.8 (49.2, 91.8) in the yips group, and 82.0 (67.6, 93.0) in the recovered group, and no statistically significant difference was noted among these groups (Z [2] = 5.115, p = 0.077, η^2^ = 0.028). The vividness scores of throwing-specific negative motor imagery in the first-person visual perspective were 24.6 (12.3, 53.3) in the control group, 73.8 (37.0, 91.8) in the yips group, and 45.1 (17.8, 68.6) in the recovered group, and a significant difference was found in the Kruskal–Wallis test among three groups (Z [2] = 23.345, p < 0.001). A large effect size (η^2^ = 0.190) was obtained. Multiple comparisons revealed that the yips group had significantly higher value than the control group (p < 0.001). The vividness scores of throwing-specific negative motor imagery in the kinesthetic perspective were 24.6 (7.4, 58.2) in the control group, 65.6 (44.7, 88.6) in the yips group, and 49.2 (24.9, 75.2) in the recovered group. The results of the Kruskal–Wallis test showed significant differences among the groups (Z [2] = 20.018, p < 0.001), and a large effect size (η^2^ = 0.160) was obtained. The post hoc test revealed a significantly higher value in the yips group than in the control group (p < 0.001).

**Fig 1:**
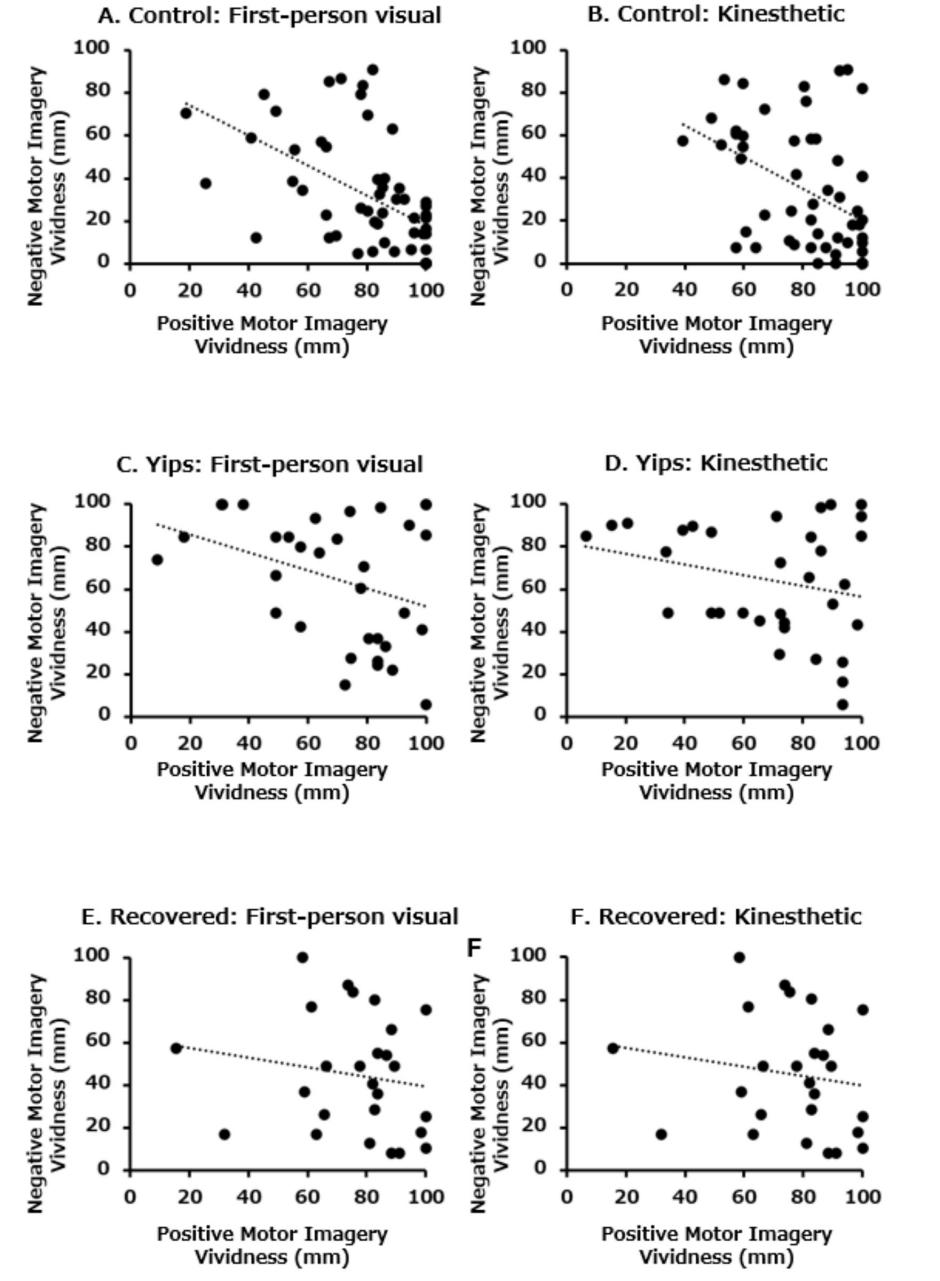
Correlation between vividness of positive and negative motor imagery. (A) First-person visual perspective in control players (p < 0.005, ρ = −0.599), (B) Kinesthetic perspective in control players (p < 0.005, ρ = −467), (C) First-person visual perspective in yips players (p = 0.155, ρ = −0.253), (D) Kinesthetic perspective in yips players (p = 0.427, ρ = −0.143), (E) First-person visual perspective in recovered players (p = 0.156, ρ = −0.287), (F) Kinesthetic perspective in recovered players (p = 0.373, ρ = −0.182).

In the control group, a significant negative correlation was found between the vividness scores of throwing-specific positive and negative motor imageries in both the first-person visual (p < 0.005, ρ = −0.599, Fig 1A) and kinesthetic (p < 0.005, ρ = −0.467, Fig 1B) perspectives. However, in both yips and recovered groups, there were no significant correlations between vividness scores of throwing-specific positive and negative motor imageries from the first-person visual (yips group: p = 0.155, ρ = −0.253, Fig 1C, recovered group: p = 0.156, ρ = −0.287, Fig 1E) and kinesthetic (yips group: p = 0.427, ρ = −0.143, Fig 1D, recovered group: p = 0.373, ρ = −0.182, Fig 1F) perspectives.

## Discussion

This study showed that the scores on the VMIQ-2, a measure of general motor imagery ability, were not significantly different among the three groups in both first-person visual and kinesthetic perspectives. However, the vividness scores of throwing-specific negative motor imagery in both perspectives were significantly higher in the yips group. These results indicate that negative motor imagery related to baseball throwing may be associated with yips symptoms.

### General motor imagery ability

First, a methodological consideration of general motor imagery ability should be mentioned. As described above, the KVIQ-2, an assessment of general motor imagery ability, includes the item “Throwing a stone into water,” which is related to ball throwing. Since the results of this item could be distinctive for players with yips, a statistical analysis independent of the KVIQ-2 total score was necessary. However, the present results showed no significant differences in this item among the three groups, as well as in the VMIQ-2 total score. Therefore, we determined it to be reasonable to include this item related to throwing motion in the VMIQ-2 total score.

A close relationship has been reported between the motor imagery ability and various pathological conditions [34–37]. Patients with brain-related disorders such as stroke or traumatic brain injury have lower motor imagery ability than the control group [45, 46]. In addition to brain damage, low back pain, amputation, and immobilization have been shown to impair the motor imagery ability [36, 47]. Thus, the capability to generate motor imagery can be impaired by various pathological conditions such as pain and neurological and musculoskeletal problems. However, the results of this study showed no significant differences for both VMIQ-2 first-person visual and kinesthetic perspectives among the three groups. The results present that the general motor imagery ability was not impaired in the first-person visual perspective or in the kinesthetic perspective in athletes with throwing yips. The reason for the discrepancy between the results of the present study and those of previous studies [34–37] is probably whether the movements used to assess motor imagery ability were actually impaired. That is, if a pathological condition such as a neurological or musculoskeletal disorder or pain is present, the patient may actually have difficulty in performing the movements used in the assessment of motor imagery ability. By contrast, yips symptoms are generally task-specific [38] and thus do not impair movements other than baseball throwing. Therefore, these differences may have contributed to the discrepancy between the results of the present study and those of previous studies.

### Vividness of throwing-specific motor imagery

As described above, the VMIQ-2 scores did not differ among the three groups. This confirms that the three groups have comparable general motor imagery ability. The vividness of positive motor imagery specific to ball throwing was not significantly different in either the first-person visual or kinesthetic perspectives among the three groups. Contrastingly, the vividness score of negative motor imagery specific to ball throwing was significantly higher in the yips group than in the control group, in both first-person visual and kinesthetic perspectives. These findings reveal that the generation of negative motor imagery related to baseball throwing may be involved in throwing yips in baseball players.

Interventions using motor imagery (mental practice) are widely used in sports [20, 21] and rehabilitation [48, 49] settings to improve motor skills. Thus, images that lead in a positive direction, i.e., to successful motor performance, are commonly used [40]. However, motor images can also be imagined in negative directions, such as failure of motor performance [40, 41]. Positive and negative motor imagery have opposing influences on motor performance [40, 50, 51]. For example, positive motor imagery improves motor performance, whereas negative motor imagery decreases it in golf putting [51] and darts throwing [39] movements. Negative motor imagery was found to be closely related to performance anxiety [41]. In addition, Williams et al. investigated whether motor imagery composed of positive and negative scripts had different effects on the interpretation of anxiety in dart throwing [52]. As a result, the latter group interpreted their anxiety to be significantly more debilitative toward dart-throwing performance than the former group. Regarding the association between yips and anxiety, we previously investigated triggers of the onset of symptoms of throwing yips in baseball players [7]; findings revealed that 43% of players with yips who reported psychological factors, such as anxiety about throwing the ball, triggered their symptoms. However, psychological factors, including anxiety, are not the only causes of the yips. In both yips and focal dystonia, a subtype of yips, symptoms may be caused by pain, injury, excessive practice, or a change in skill [53]. In such cases, anxiety may exacerbate the symptoms [38, 54]. Thus, anxiety and negative motor imagery can have both direct and indirect influences on yips symptoms. That is, the anxiety about throwing can generate negative motor imagery, which directly causes yips symptoms. Conversely, yips symptoms may cause poor ball control, which results in a vicious cycle of worsening yips symptoms by increasing anxiety about ball throwing and negative motor imagery. Thus, the vividness of negative motor imagery should be assessed in all players with yips because it may directly or indirectly influence yips symptoms. In addition, interventions addressing psychological aspect, such as anxiety, which are a potential cause for the generation of negative motor imagery, may be necessary to alleviate or prevent the symptoms of yips.

In this study, the vividness score of positive motor imagery specific to ball throwing tended to be lower in the yips group for both visual (p = 0.082) and kinesthetic (p = 0.077) perspectives; however, no significant differences were found. Conversely, the vividness score of negative motor imagery was significantly higher in the yips group than in the control group in both first-person visual and kinesthetic perspectives, yielding a large effect size. This discrepancy between the two measures of vividness of motor imagery related to throwing may be due to the lack of significant correlation between positive and negative motor imagery vividness in the yips and recovery groups, unlike in the control group, between whom there was a significant negative correlation. These results suggest that the two types of throwing-related motor imagery vividness are mutually independent measures in athletes who experienced the yips. Therefore, we consider that practitioners working with yips players should evaluate both types of motor imagery vividness. Whether interventions using positive motor imagery can reduce negative motor imagery and yips symptoms is a particularly intriguing question for future research.

In this study, no significant difference in the vividness of throwing-specific negative motor imagery was found between the recovered group and the control group. This indicates that the improvement in yips symptoms may have been accompanied by a reduction in the vividness of negative motor imagery related to throwing. However, the vividness of throwing-specific negative motor imagery in the recovered group was not significantly different from that in the yips group. These results might suggest that improvement in yips symptoms does not restore the vividness of negative motor imagery. To our knowledge, no studies to date have compared the characteristics of players whose yips symptoms have improved with those who continue to have symptoms. This comparison may provide important insights to the improvement of yips symptoms, and further research is warranted.

This study has several limitations. The participants of this study were only male collegiate baseball players with an average of >10 years of baseball experience. Therefore, whether the results of this study would apply to professional or high school baseball players with different baseball skills or to women is unclear. This should be taken into account when interpreting the present findings. In this study, we evaluated the general motor imagery ability and the vividness of motor imagery specific to baseball throwing. However, whether other aspects of motor imagery ability, such as mental chronometry [55], which measures the time required to imagine a certain movement, are impaired in baseball players with the throwing yips remains unclear. This is relevant for understanding the temporal nature of motor imagery in athletes with the yips and requires further study.

## Conclusions

This study reveals that while the general motor imagery ability among athletes experiencing yips did not differ, negative motor images were more vividly imagined. This may contribute to the development of throwing yips symptoms in baseball players and could lead to a vicious cycle. We propose that interventions addressing psychological aspects, such as anxiety, which could be responsible for the generation of negative motor imagery, may be necessary to alleviate or prevent the symptoms of yips.

## Supporting information

S1 Checklist STROBE checklist. (DOCX)

S1 Table The complete raw dataset of this study. (XLSX)
